# At-Home Red Light Therapy Devices: Promotion and Recommendation Patterns on Social Media in the Context of Limited Evidence

**DOI:** 10.7759/cureus.103274

**Published:** 2026-02-09

**Authors:** Taylor Merkle, Luke Tomczak, Khai Do, Michael Aji, Erum Ilyas

**Affiliations:** 1 Dermatology, Drexel University College of Medicine, Philadelphia, USA; 2 Dermatology, Pennsylvania State University College of Medicine, Hershey, USA

**Keywords:** low-level light therapy, photobiomodulation, red light, red light therapy, social media

## Abstract

The popularity of red light therapy (RLT) has grown substantially, with social media playing a significant role. The objective of this study was to characterize how RLT is promoted on social media and to assess whether these claims align with current evidence-based dermatologic applications by examining creator credentials, promoted device characteristics and costs, claimed indications, and patterns of content reach that may contribute to acceptance of unverified health-related claims. After exclusions, 132 posts were analyzed with an overall potential reach of 47.5 million followers. While non-credentialed authors created 64.4% of posts compared to 18.2% of physicians, the overall reach of physician posts was 38.9% based on the total number of followers. Red light (RL) + near-infrared (nIR) devices were most commonly recommended (63.7%), followed by multiwavelength devices (MWD) (23.4%). RL-only devices were recommended by 1.6% of posts. Median device price varied, with non-credentialed posts recommending lower-priced devices ($347), followed by physicians ($469), and then other advanced degree and licensed professionals ($629). A significant association was found between platform and credential (χ² (2) = 25.85, p < 0.0001), with TikTok posts predominantly from non-credentialed accounts (87.7%) and Instagram being more mixed. Credentialed accounts were more likely to recommend RL + nIR over MWD (χ² = 6.80, p = 0.0091). "Skin" was the most consistently referenced benefit category at 88.6%, with physicians focused on anti-aging and acne benefits (66.7% and 20.8%) and non-physicians on a broader range of skin targets. While vague references to "research" and "studies" were made by most posts, only 8.3% of posts provided peer-reviewed journal articles as support. There was a discrepancy in terms of energy output from promoted at-home devices, which remain untested, compared with the specifications found in these references. Overall, the claimed benefits of RLT and promotional at-home devices on social media are often well beyond substantiated evidence, which may leave patients with unrealistic expectations. Factors influencing trust in promotional claims may vary due to the limited ability of platforms to verify credential authenticity. Although physicians make up a smaller percentage of content, their overall potential reach provides the opportunity to enhance evidence-based guidance rather than reinforce commercially driven messaging.

## Introduction

At-home red light therapy (RLT) devices are marketed for a wide range of aesthetic and medical benefits, with a global market estimated at $361.5 million in 2024, growing annually, with social media playing a role in their rising popularity [1,2]. A cross-sectional analysis study found that 90% of surveyed individuals were willing to purchase RLT devices, with 60.4% of respondents having learned about it through social media [3]. While photobiomodulation research supports some applications with targeted device specifications, there remains a significant gap between the claims made, supporting evidence, and specific devices marketed to consumers for at-home use [1,4]. This discrepancy has contributed to widespread promotional claims and misinformation about RLT on social media, which has not been systematically characterized.

The objective of this study was to assess how RLT is promoted on social media and if these claims are representative of current evidence-based dermatologic applications, with particular attention to factors influencing unverified health-related claims. Specifically, we evaluated (i) professional credentials of creators; (ii) types, costs, and technical wavelength parameters of promoted at-home RLT devices and the claimed benefits and indications for their usage; and (iii) patterns of content reach and potential factors contributing to patient acceptance of unverified claims, such as misleading promotional framing. Findings will help clinicians understand the source of patient information and support informed patient-provider discussions. 

## Materials and methods

To assess social media platforms for RLT content, new Instagram and TikTok accounts were created to minimize the influence of preexisting algorithms on content feeds. Four reviewers participated in a structured data collection process, each reviewing 35 posts between June 30 and July 18, 2025, on specific days to ensure consistency given the potential for trends to influence feeds. Search terms "red light therapy", "red light", "red light devices", and "red light recommendations" were entered into discovery/search pages under the "Top" or "For You" category. After each search, the first appearing post was selected for review, and this process was repeated for each search term until a total of 35 posts were collected by each individual reviewer. Duplicate posts were not recorded by individual reviewers, whereas duplicate posts between different reviewers were excluded.

Reviewers documented the credentials of the post author, number of followers, device brand name, price, and specifications, and post caption and claims, along with supporting citations. Credentials of content creators were recorded: (i) physician, (ii) other advanced degree or licensed professional, and (iii) no credentials. Posts were excluded if they were non-English, duplicates, photo-only without associated text or captions, or spa promotions. To validate data collection, a random sample of five posts per reviewer group was cross-checked for inter-rater reliability. Reviewer agreement was high across variables, with rare discrepancies including classification of post type for benefit claim categories and definition of exclusion criteria, specifically ad-related spa and promotional posts with only photos and no text among one reviewer; these were resolved through consensus with a faculty advisor. 

Post captions were compiled and entered into ChatGPT (OpenAI, San Francisco, USA) to generate a list of benefit categories based on commonly used phrases and references. Benefit categories were then reviewed and condensed into a table format with submetrics based on clinical relevance determined by the human reviewers of this study. Reviewers then assigned each post individually to one or multiple categories based on the post content. Posts were subsequently manually tallied by category, with submetrics tallied separately. Simple statistics and chi-square tests were performed to analyze data, with p < 0.05 designated as statistically significant.

## Results

A total of 140 posts were collected, with 132 posts analyzed (75 Instagram, 57 TikTok) after exclusions, with a potential reach (according to total number of followers) across all platforms exceeding 47.5 million. While 64.4% of posts came from non-credentialed accounts, followed by physicians (18.2%), based on follower count, physician posts accounted for 38.9% of social media reach. The other advanced degree or licensed professional category included podiatrists, Doctor of Philosophy (PhD) holders, pharmacists, chiropractors, physical therapists, nutritionists, integrative health professionals, optometrists, naturopaths, Master of Public Health (MPH) holders, registered nurses (RNs), and aestheticians (Table 1). 

**Table 1:** Social media posts from Instagram and TikTok categorized based on credentials and tallied to include number of followers reached, wavelengths of devices recommended, median pricing, and research support claims. Data are presented as tallied counts (N), medians (range), and percentages (%) of the most frequent recommendation based on the total number of devices recommended within each credential category. ^1^Multiwavelength devices (MWD) included: (UVA + UVB + RL + nIR); (UVB + YL + RL + nIR); (YL + RL + nIR); (BL + GL + RL); (GL + RL + BL + nIR); (RL + BL+ GL + YL + PL + OL + CL); (RL + nIR + dIR + BL + GL + PL + CL + YL + WL); (RL + nIR + YL + BL). RL = red light; nIR = near-infrared; dIR = deep infrared; BL = blue light; YL = yellow or amber light; GL = green light; PL= purple light; OL = orange light; CL = cyan light; WL = white light; USD = United States Dollar

	Physician (N)	Other Advanced Degree or Licensed Professional (N)	No credentials (N)	Totals (N)
Total Instagram posts	18	22	35	75
Total TikTok posts	6	1	50	57
Total reach of followers	18,484,800	13,111,668	15,915,121	47,511,589
RL devices	0	0	2	2
RL + nIR (mask, panel, wand) devices	13	24	39	76
RL + nIR (belts, nasal) devices	0	0	3	3
RL + nIR +/- dIR or BL devices	5	2	7	14
MWD^1^ devices	3	3	23	29
No specific devices recommended	1	2	15	18
No recommendations due to insufficient data	1	0	0	1
Most frequent recommendation: RL + nIR (%)	13 (61.9%)	24 (82.8%)	42 (56.8%)	79 (63.7%)
Median price in USD (range)	469 (348-159,500)	629 (189-3,999)	347 (7-159,500)	469 (7-159,500)
Peer-reviewed journal articles	3	4	4	11

Red light (RL) + near-infrared (nIR) devices were most commonly recommended (63.7%), followed by multiwavelength devices (MWD) (23.4%), with the least as RL-only devices (1.6%). MWD included a broad spectrum of light from 280 nm to 1100 nm, inclusive of intermediate light sources. About 79% of MWD recommendations came from non-credentialed accounts. Although 13.6% of posts did not include a specific device, shoppable links for numerous devices were found in user profiles. Only one post specifically stated that there was a lack of data supporting at-home RLT products. Median device price varied, with non-credentialed posts recommending lower-priced devices ($347), followed by physicians ($469), and then other advanced degree or licensed professionals ($629) (Table 1). 

There was a significant association between platform and credential (χ² (2) = 25.85, p < 0.0001, Cramer’s V = 0.44), with TikTok posts predominantly from non-credentialed accounts (87.7%) and Instagram being more mixed. Credentialed accounts were more likely to recommend RL + nIR over MWD (χ² = 6.80, p = 0.0091, φ = 0.25). 

Few posts (n = 11) made reference to specific research articles, while most posts made vague references to "evidence," "research," "science," "NASA," or personal experience (Table 1). The small number of studies cited by these posts showed inconsistent findings with some potential for RL - mostly for skin use - based on specific device parameters, but little data were available to support broader claims. Additionally, no consistent benefits were noted for nIR in the articles cited. Aside from similar wavelengths of visible light referenced, articles frequently did not match the benefits promoted or energy outputs and platforms of the devices recommended by posts [1,4]. 

"Skin" was the most consistently referenced benefit category at 88.6%, with physicians focused on anti-aging and acne benefits (66.7% and 20.8%) and non-physicians on a broader range of skin targets. Aside from "skin," other advanced degrees and licensed professional accounts tended to skew toward physiologic categories such as metabolic, performance, and sleep, while non-credentialed accounts claimed broad yet less focused benefits across categories (Table 2). 

**Table 2:** Claimed benefits by category/subcategory per post tallied based on number of post mentions. Bolded phrases indicate categories with subcategories italicized below. Data are presented as tallied counts (N) and percentages (%) of total posts within each credential category. Any single post that discussed multiple benefits was tallied individually per category. EBV = Epstein-Barr virus; staph = staphylococcal infection

	Physician (N)	Other Advanced Degrees/ Licensed Professionals (N)	No Credentials (N)	Totals (N)
Total number of posts	24	23	85	132
Skin (%)	23 (95.8%)	20 (87%)	74 (87.1%)	117 (88.6%)
*Acne *(%)	5 (20.8%)	3 (13%)	5 (5.9%)	
*Actinic damage* (%)	1 (4.2%)	2 (8.7%)	6 (7.1%)	
*Anti-aging (fine lines, collagen, elastin, wrinkles) *(%)	16 (66.7%)	13 (56.5%)	48 (56.5%)	
*Herpes* (%)	1 (4.2%)	0 (0%)	0 (0%)	
*Hyperpigmentation/melasma* (%)	3 (12.5%)	0 (0%)	10 (11.8%)	
*Inflammation, redness* (%)	4 (16.7%)	12 (52.2%)	29 (34.1%)	
*Radiation dermatitis *(%)	1 (4.2%)	0 (0%)	0 (0%)	
*Scar *(%)	4 (16.7%)	2 (8.7%)	6 (7.1%)	
*Wound healing *(%)	3 (12.5%)	9 (39.1%)	11 (12.9%)	
*Hair loss* (%)	3 (12.5%)	5 (21.7%)	11 (12.9%)	
*Other (burns/sunburns, EBV, Lyme disease, staph, tinea, mast cell, rosacea, lymphatic drainage, parasites/mold, psoriasis, vitiligo, eczema, sensitive skin) *(%)	0 (0%)	8 (34.8%)	17 (20%)	
Cellular/metabolic, mitochondria/energy (%)	3 (12.5%)	13 (56.5%)	19 (22.4%)	35 (26.5%)
Muscle recovery, performance (%)	4 (16.7%)	10 (43.5%)	17 (20.0%)	31 (23.5%)
Sleep (circadian rhythm, melatonin) (%)	2 (8.3%)	9 (39.1%)	10 (11.8%)	21 (15.9%)
Circulation (%)	1 (4.2%)	7 (30.4%)	11 (12.9%)	19 (14.4%)
Bone/joint health (%)	4 (16.7%)	5 (21.7%)	7 (8.2%)	16 (12.1%)
Hormone (%)	2 (8.3%)	6 (26.1%)	6 (7.1%)	14 (10.6%)
*Thyroid *(%)	0 (0%)	3 (13.0%)	2 (2.4%)	
*Blood sugar, menstrual cycle, testosterone, limit cortisol release* (%)	0 (0%)	3 (13.0%)	2 (2.4%)	
Chronic pain (%)	2 (8.3%)	4 (17.4%)	8 (9.4%)	14 (10.6%)
Mental health (depression/anxiety, seasonal affective disorder, mood) (%)	2 (8.3%)	3 (13.0%)	6 (7.1%)	11 (8.3%)
Cognition, nervous system health, headaches, migraines (%)	1 (4.2%)	5 (21.7%)	4 (4.7%)	10 (7.6%)
Fat reduction/weight loss, gut health (%)	0 (0%)	1 (4.3%)	6 (7.1%)	7 (5.3%)
Immune health (%)	0 (0%)	2 (8.7%)	5 (5.9%)	7 (5.3%)
Eye health, age-related vision loss, myopia (%)	1 (4.2%)	4 (17.4%)	1 (1.2%)	6 (4.5%)

## Discussion

With reports that over half of adult TikTok users in the United States regularly receive news from social media, and 45% of those aged 18-34 years trusting the average person over a physician for medical advice, it is noteworthy that 64.4% of social media posts evaluated in our study originated from non-credentialed sources [5,6]. Given the usage of social media as a growing primary source for health information, perceived trust may therefore be influenced by follower count, repetitious exposure to promotional benefit claims, and professional-appearing labels rather than verified clinical expertise. 

Credentialed versus non-credentialed accounts in this study were classified based on publicly stated degrees, professional titles, or licensure information available within account profiles, reflecting how expertise is presented to consumers on these platforms with limited ability to verify status or claims. The skew toward non-credentialed accounts was strongest on TikTok (87.7%), whereas Instagram was more mixed. A study assessing popular dermatology-related posts on TikTok similarly found only 30% representation from board-certified dermatologists for profession-specific hashtags, indicating a substantial proportion of health-related content is produced by individuals without formal medical training [7].

Without the ability to verify credential status, consumers may rely on account authenticity - represented by a blue check mark on Instagram or TikTok - as a potential misclassified signal for trust, granted solely based on platform-specific requirements, not medical credentials. Paired with the repeated messaging of similarly claimed benefits for promotional devices across multiple creator posts, perceived authority may be reinforced when encountering these misleading designations. The resulting ambiguity in content reliability facilitates the acceptance of unsubstantiated advice and recommendations from non-credentialed creators or creators with misleading credential claims. Interestingly, although physician posts accounted for only 18.2% of content across both platforms, the potential reach of these posts was 38.9% of the total follower count, suggesting a larger potential impact on advice offered.

A prior published study reported that most respondents (48.9%) were willing to pay between $101-$300 for these devices, with fewer (10.1%) inclined to pay higher price ranges upward of $500 [3]. Our study revealed a wide range in costs for RLT devices from $7 to $159,500; thus, consumers may rely on trusted recommendations when making financial decisions concerning these products. Non-credentialed accounts promoted devices with a median price of $347; however, these were often MWD with no clearly supported added benefits at differing wavelengths of light and risk of diluted overall RL output. One study evaluating simultaneous and sequence emissions of varying wavelengths in low-powered and light-emitting diode (LED) devices found modifications in the effective irradiance per band, which did not match with studies reporting single wavelengths [8]. This demonstrates that biological responses are wavelength-specific and nonlinear, making it unreliable to assume that combining wavelengths yields additive or equivalent effects. Furthermore, home-use devices typically operate at lower power outputs than professional systems for safety reasons, as patients use these products without direct medical oversight, emphasizing the limited generalizability of existing research to at-home RLT applications [9]. Product availability and commission-based sales may influence content, with only 1.6% of posts recommending RL alone. These findings suggest that social media users may therefore be influenced by repetitive messaging, perceived credential verification and authority status, and promotional claims fostering trust in less effective devices for purchase based on price rather than science-based evidence.

While RLT in studies shows at most a modest indication for specific benefits, efficacy and safety rely heavily on device parameters such as wavelength, energy delivered, distance from the skin, duration and frequency of use, as well as potential considerations for skin type [1,4]. A review of various at-home devices for indications of acne, photoaging, scars, psoriasis, and hair regrowth found limited evidence of effectiveness, with most studies being small, industry-sponsored, uncontrolled trials lacking long-term follow-up [10]. Extrapolating findings from studies to apply to at-home units is likely an overreach and is further limited by safety concerns. With a lack of uniform regulations pertaining to at-home devices, many devices are readily cleared by the U.S. Food and Drug Administration (FDA) and given an "FDA-cleared" label, designating only a low risk to the public and eliminating the need for clinical trials on new devices. However, this distinction can lead to misinterpretation regarding efficacy [11,12]. Interestingly, only one post from a physician specifically noted that no recommendations on specific devices could be made by the post, given the lack of data available for at-home RLT use. 

This study has several limitations, including its capture period for posts that may be impacted by trends, as well as restriction to only two social media platforms. The preferred social media platform by users can vary based on demographics and the type of content sought by users. Reliance on the follower count for post "reach" is also limited by algorithms and may potentially be lower or higher based on interest. Additional engagement metrics to measure content influence, such as shares, likes, and comments, were not reported in this study. This study focused on the text content of posts, excluding visual content analysis, which may be highly influential on social media. Future studies could consider the impact of visual cues from posts and the cumulative impact of viewing multiple posts with aligned content that reinforce poorly substantiated claims.

## Conclusions

While RLT shows promise for certain dermatologic applications, efficacy is highly dependent on the use of specific wavelengths of light, power, and dose duration parameters that vary by treatment goal and remain clinically untested in at-home devices. Clinicians should therefore be aware of the social media portrayal of these devices, as well as beyond substantiated scientific evidence, with a large proportion of claims made by non-credentialed creators. With limited availability to readily verify credential status and claims, the promotion of at-home devices may be influenced by factors such as repetitive exposure to posts with similar claims and misplaced trust in authenticity signaling. Although physicians make up a smaller percentage of content, the overall higher potential reach based on follower count highlights a critical opportunity for content to reflect accuracy and nuance to set patient expectations as opposed to simply promoting further product sales. 
